# Designing a Culturally Relevant Digital Skin Cancer Prevention Intervention for Hispanic Individuals: Qualitative Exploration

**DOI:** 10.2196/56939

**Published:** 2024-09-12

**Authors:** Zhaomeng Niu, Yonaira M Rivera, Carolina Lozada, Shawna V Hudson, Frank J Penedo, Sharon L Manne, Carolyn J Heckman

**Affiliations:** 1 Department of Health Informatics Rutgers School of Health Professions Piscataway, NJ United States; 2 School of Communication and Information Rutgers University New Brunswick, NJ United States; 3 Cancer Prevention and Outcomes Data Support Rutgers Cancer Institute New Brunswick, NJ United States; 4 Department of Family Medicine and Community Health Rutgers Robert Wood Johnson Medical School New Brunswick, NJ United States; 5 University of Miami Sylvester Comprehensive Cancer Center Miami, FL United States; 6 Department of Medicine Robert Wood Johnson Medical School Rutgers Cancer Institute New Brunswick, NJ United States

**Keywords:** skin cancer, prevention, Hispanic, sun protection, skin self-examination, intervention, qualitative, interviews, health care provider, community leader, mobile-based

## Abstract

**Background:**

In the past 2 decades, melanoma incidence among Hispanic individuals has risen by 20%. The mortality rate of Hispanic individuals is higher than that for non-Hispanic White individuals. Skin cancer can largely be prevented with regular sun protection, and skin cancer outcomes can be improved through early detection, for example, by skin self-examination. Alarmingly, Hispanic individuals are less aware of the symptoms and harms of skin cancers, tend to have misperceptions regarding the risks and benefits of skin cancer prevention behaviors, and engage in less sun protection behaviors than non-Hispanic White individuals.

**Objective:**

This study aimed to use a community-engaged approach and conduct both group and individual interviews among Hispanic individuals and relevant key stakeholders to explore the potential design of a mobile-based skin cancer prevention intervention for Hispanic individuals.

**Methods:**

This study used a qualitative design (focus groups and individual interviews). Participants were recruited from local community organizations’ social media, local events, and contact lists (eg, email). Zoom interviews were conducted to examine whether Hispanic individuals would be interested in a mobile-based skin cancer intervention and to explore their preferences and suggestions to inform skin cancer prevention intervention design.

**Results:**

Five focus groups (2 in Spanish and 3 in English) among self-identified Hispanic individuals (n=34) and 15 semistructured, in-depth individual interviews among key stakeholders (health care providers and community leaders; eg, dermatologist, nurse practitioner, licensed social worker, and church leader) were conducted. The main themes and subthemes emerging from the group discussions and individual interviews were organized into the following categories: intervention platform, delivery frequency and format, message design, engagement plan, and activities. WhatsApp and Facebook were identified as suitable platforms for the intervention. Messages including short videos, visuals (eg, images and photographs), and simple texts messages were preferred. Recommendations for message design included personalized messages, personal stories and narratives, culturally relevant design (eg, incorporating family values), and community-trusted sources. Potential engagement and retention recommendations were also discussed. Additional details and exemplar quotes of each theme and subtheme are described.

**Conclusions:**

This study provides important insights and directions for the design of a mobile, digital skin cancer intervention to modify Hispanic individuals’ sun protection and skin self-examination behaviors to help improve skin cancer outcomes. Insights gathered from community leaders and health care providers provided valuable additions to the community-derived data. Leveraging popular digital platforms among Hispanic individuals such as WhatsApp or Facebook could be a promising approach to skin cancer prevention. Recommendations from the community included the use of concise videos, illustrative images, clear text messages, tailored communications, narratives featuring personal experiences, designs that reflect cultural significance, and information from sources that are trusted by the community, which provided useful strategies for future intervention design among Hispanic individuals.

## Introduction

The Hispanic population is the fastest growing ethnic group in the United States [1]. Hispanic individuals account for 18.5% of the nation’s population and have become the largest racial or ethnic minority group [2]. Hispanic individuals tend to experience disparities in health care and medical services, usually have lower levels of health literacy, have greater fatalistic beliefs about cancer prevention [3], and have worse preventive health behaviors [4-7].

Skin cancer is the most common cancer in the United States [8,9]. Melanoma has the highest fatality rate of all skin cancers [10]. Melanoma incidence among Hispanic individuals has risen by 20% in the past decade [11]. Although the Hispanic population has lower incidence rates of skin cancers than non-Hispanic White individuals, the mortality rate among Hispanic individuals is higher [11]. Hispanic individuals diagnosed with melanoma tend to be younger, diagnosed at more advanced stages with thicker tumors, and have worse 5-year survival rates than non-Hispanic White individuals [12-15]. Yet, despite these disparities, skin cancer prevention and education efforts are understudied among the Hispanic population [12]. Alarmingly, Hispanic individuals are less aware of the symptoms and harms of skin cancers, tend to have misperceptions regarding the risks and benefits of skin cancer prevention behaviors, and engage in less sun protection behaviors than non-Hispanic White individuals, and only 18% of Hispanic individuals report ever having done a skin self-examination [16]. In addition, due to the increase of acculturation of Hispanic individuals in the United States, their engagement in sun safety behaviors is poorer than that of non-US Hispanic individuals. Therefore, an increasing effort to raise awareness about sun protection behaviors and skin self-examination behaviors is needed to address the existing gap of skin cancer research among Hispanic individuals in the United States.

In 2021, almost 100% of US Hispanic individuals owned a cell phone, and 85% owned a smartphone [17]. Moreover, Hispanic individuals are large consumers of social media and report using these platforms to engage with and use health information [18]. Mobile-based digital health interventions may reduce several structural barriers (eg, cost and distance) to health care, are sometimes more acceptable to underserved populations than traditional media or in person programs [19,20], and could be a useful approach to prevent cancer among Hispanic individuals. The literature on culturally relevant and effective skin cancer intervention for Hispanic individuals is notably scarce, and providing formative research results could help advance intervention development for this group. This study collected qualitative data from key Hispanic stakeholders and community members to examine whether Hispanic individuals would be interested in a mobile-based skin cancer intervention and to explore their preferences and suggestions to inform skin cancer prevention intervention design.

## Methods

### Study Design

Nested within a larger mixed methods study aiming to develop a skin cancer intervention among Hispanic individuals, this qualitative study identified preferred design features (eg, content, delivery mode) of a mobile-based and culturally targeted skin cancer intervention for Hispanic individuals. Hispanic culture plays an important role in impacting health [21]; thus, developing health content to be compatible with Hispanic culture by addressing cultural values, behaviors, worldview, and belief systems is critical [22]. We interviewed community members through focus group discussions and key stakeholders (ie, community leaders and health care providers) through one-on-one interviews. The research team followed the recommendations from McCracken [23] to develop an interview guide for both individual and group interviews, in which a comprehensive literature review was conducted first and then an iterative process for data collection, interview guide modifying, and data analysis was used.

### Recruitment

Our study team collaborated with Rutgers University’s Office of Community Outreach and Engagement (COE), which provides research and outreach support to reduce the cancer burden. Through the COE office, we connected with 3 community organizations to recruit Hispanic participants for the focus group discussions with Hispanic adults and individual key stakeholder interviews (eg, a local outdoor laborer organization). Community members were able to be efficiently interviewed in groups, whereas community leaders and health care providers required individual interviews due to their demanding schedules.

For focus group participants, we recruited from community organizations’ social media, local events, and contact lists (eg, email). In addition to recruitment from community organizations, we distributed flyers at local events and fairs (eg, school fair and health fair) and posted recruiting study messages on social media platforms including Reddit groups, Craigslist, Facebook pages and groups, Twitter (subsequently rebranded as X), and Instagram in English and Spanish. We also used snowball sampling starting from community organizations and university hospitals and clinics to reach out to key stakeholders, including community leaders and health care providers. For health care providers, the study team reached out to them via emails and phone calls. Our study team comprised bilingual researchers and staff members to help with all aspects of the recruitment and analysis process.

#### Focus Groups

Inclusion criteria of Hispanic adults recruited for the focus groups included (1) self-reporting as Hispanic; (2) being 18 years of age or older; (3) having no personal history of skin cancer; (4) having 1 or more skin cancer risk factors [24]; and (5) either not engaging in sufficient sun protection behaviors or not having had a skin self-examination conducted in the past 3 months. This was defined as mean score of less than 4 on the sun protection behaviors [25], which assessed wearing sunscreen, long-sleeved shirt, long pants, and sunglasses and staying in shade or under an umbrella in the last 3 months (1=never, 5=always).

Hispanic individuals who were interested in the study completed a short web-based screening survey in English or Spanish to determine their eligibility and provide contact information and availability to take part in a focus group. Eligible respondents were contacted by the study team to confirm study interest and group assignment based on their availability. Two email reminders were sent to the respondents before their scheduled focus group. All study materials were available in both English and Spanish.

#### Individual Interviews

To be eligible for key stakeholder interviews, community leaders must have self-identified as Hispanic community leaders (eg, as a church leader or the director of a community organization). For individual interviews with community leaders identified either from the COE office or snowball sampling via public websites or channels, the study team emailed them individually for scheduling. Health care providers were eligible for this study if they had experience serving the Hispanic community.

### Data Collection

Data collection occurred between March and July 2023. All focus groups and individual interviews were conducted virtually via Zoom (Zoom Video Communications, Inc). Interview sessions were facilitated by 2 members of the research team, and interview notes were taken during interviews. Participants were asked questions regarding skin cancer among Hispanic individuals, sun protection and skin examination among Hispanic individuals, their social media use, and their opinions regarding a mobile-based skin cancer intervention among Hispanic individuals. We conducted 5 focus groups (lasting 60-90 minutes with 5-11 participants per group) and 15 individual interviews (45-60 minutes per interview) to reach thematic saturation [26]. Interviews were conducted in the participant’s preferred language. English interviews were transcribed via Zoom’s autotranscription function and reviewed by a research team member; Spanish interviews were transcribed and translated by a professional transcription service.

### Ethical Considerations

This study was approved by Rutgers University’s Institutional Review Board (Pro2021001562; Pro2022000533). The institutional review board–approved informed consent form was sent to the participants before the interviews, and oral consents were obtained at the beginning of the interviews. Participants were assigned a study ID, and the qualitative data were stored in the university password-protected cloud drives for privacy and confidentiality protection. Participants received up to US $70 in electronic Amazon gift cards as compensation for participating.

### Statistical Analysis

A thematic analysis was conducted on all interview transcripts to capture insights and emerging themes related to skin cancer intervention design preferences. We used Glazer’s emerging design, which emphasizes that it is important to allow themes or categories emerging from the data instead of using preexisting themes or categories to frame the data or the codes [27]. Four team members first reviewed transcripts to become familiar with the data and create a preliminary codebook based on their analysis of the data. Each coder was assigned 1 transcript to code in full using ATLAS.ti. When finished, the same transcript was assigned to another coder for double coding to ensure consistency of the coding agreement. After double coding 1 transcript, all coders met to discuss questions during coding, discrepancies on the codes, and new codes emerging from the transcript and to decide whether expanding the code list was necessary. These discussions occurred across several weekly meetings, during which they identified overlapping codes, refined code definitions, excluded unnecessary codes, and included new emerging codes. When there were no new codes emerging from the weekly meetings, the coders started to apply the final code list to the rest of the interview transcripts while creating their coding memos. The coding memos documented emerging and reoccurring themes from the transcripts, coding thoughts and questions from the coding process, and representative quotations. During these meetings and deliberations, the research team was able to successfully finalize the themes emerging from the coding memos.

## Results

### User Statistics

Table 1 summarizes the demographic information of focus group participants and key stakeholders. Five focus groups (2 in Spanish and 3 in English) were conducted among self-identified Hispanic individuals (n=34), with 71% (n=24) born in the United States and 24% (n=8) born in Mexico. Participants had an average age of 33.44 (SD 12.4) years and mainly reported having received a college degree or higher (21/34, 62%), having an average income of US $50,000 or higher (20/34, 59%), and being single or divorced (20/34, 59%).

**Table 1:** Demographic characteristics of New Jersey Hispanic participants (n=34) and stakeholders (n=15) in focus groups and interviews on skin cancer prevention intervention design, 2023-2024.

Demographic characteristics				Value
**Hispanic participants (n=34)**				
	Age (years), mean (SD)			33.44 (12.4)
	**Sex, n (%)**			
		Female	17 (50)	
		Male	17 (50)	
	**Place of birth, n (%)**			
		United States (including Puerto Rico)	24 (71)	
		Outside of the United States	10 (29)	
	**Education, n (%)**			
		Less than high school	2 (6)	
		High school graduate	3 (9)	
		Completed some college	8 (24)	
		College degree or associate’s degree	14 (40)	
		Graduate degree	7 (21)	
	**Total household income (US $), n (%)**			
		<25,000	10 (29)	
		25,000-49,999	4 (12)	
		50,000-74,999	9 (26)	
		75,000-149,999	7 (21)	
		≥150,000	4 (12)	
	**Marital status, n (%)**			
		Married or living with a partner	14 (41)	
		Single	15 (44)	
		Divorced or separated	5 (15)	
**Stakeholders (n=15)**				
	Age (years), mean (SD)			43 (22.6)
	**Sex, n (%)**			
		Female	13 (87)	
		Male	2 (13)	
	**Role, n (%)**			
		Dermatologist or oncologist	2 (13)	
		Nurse practitioner	4 (27)	
		Licensed social worker	2 (13)	
		Clinical coordinator	2 (13)	
		Community leaders	5 (33)	

In addition, 15 semistructured in-depth individual interviews (female: n=13, 87%; male: n=2, 13%; ages ranging from 25 to 72 years) were conducted with key stakeholders, including health care providers and community leaders. One dermatologist, an oncologist, 4 nurse practitioners, 2 licensed social workers, 2 clinical coordinators at the university, and 5 community leaders (eg, organization leader, church leader) were interviewed.

Interview example questions are shown in Textbox 1. Main themes and subthemes emerging from the group interviews and individual interviews were organized into the following categories: intervention platform, delivery frequency and format, message design, and engagement plan and activities. These themes are discussed in detail, with exemplary quotes for each subtheme shown in Table 2.

**Interview guide example questions for skin cancer prevention intervention design with New Jersey Hispanic individuals, 2023-2024.**

**Focus groups**

1. What can you tell me about skin cancer?
  - What can you tell me about skin cancer and the Hispanic community?
  - What is the association between skin cancer and the Hispanic community?
  - What other health topics do you think are important for the Hispanic community?
2. What are the reasons that you protect your skin from the sun? (Be careful about giving examples: expensive, care about skin/aging)
  - What helps you protect your skin? What makes it hard?
  - What are the downsides of sun protection?
3. What things make it easy for you to check your skin? What things make it hard for you to check your skin?
  - What are the downsides of skin examination?
4. We want to create an educational program to help Hispanic individuals protect and check their skin more, because mortality rate of skin cancer is higher among Hispanic individuals than White individuals. What would you want to see in a skin cancer prevention educational program? A program is that we give you some information, could be though different platforms such as app, website, social media, etc. The program is also interactive, and we can have some interactions with you.
  - What kind of content and information would you want to see in this program?
  - How would you like to see this information presented? Videos, pictures, text, live discussions, etc.?
  - Please give me some examples of what that would look like.
  - What cultural aspects important to Hispanic individuals do you think should be a part of the messages in this program?
  - What language do you prefer to use for this program?

**Individual interviews^a^**

- What have you heard from the Hispanic community about sun safety and sun protection? Is this important among the Hispanic community? Why?
  - How do your patients usually protect their skin from the sun?
- What have you heard about skin cancer examination from your Hispanic patients or clients?
  - Do you believe this is important to Hispanic individuals? If it is important, why would it be?
  - Do your patients usually check their skin for any changes or unusual growths? If so, how do they do it?
- What is your experience communicating with your Hispanic patients/clients? Especially with any media or social media platform?
  - How do you communicate with your patients/clients? What social media do you use to communicate with your Hispanic patients?
  - What do you think about using apps or websites for health behavior change among Hispanic individuals?
- What do you think would be good to include in a skin cancer prevention educational program for Hispanic individuals or what content would benefit them the most?
  - What kind of content and information? Frequency? Incentive?
  - What cultural aspects important to Hispanic individuals do you think should be a part of the messages in this program?
  - What other health messages or information (in addition to skin cancer information) do you think we can include in this program to make it more interesting and relevant to the Hispanic community?
  - What kind of messages can help promote communication (family communication/interpersonal communication) regarding skin cancer prevention within the Hispanic community?

^a^Interview guides are developed in an iterative way and in both Spanish and English.

**Table 2:** Emergent themes, subthemes, and example quotes from interviews and focus groups on skin cancer prevention intervention design for New Jersey Hispanic individuals, 2023-2024.

Themes and subthemes			Quotes
**Intervention platform preference**			
	WhatsApp		“I use WhatsApp for many things. I have groups with family members that sometimes want to make decisions about family things, and we make videos, and we all talk together about it. I like it a lot because it is more practical, and many people use it. I'm always seeing what people post, what my family posts; sometimes I make invitations for parties, birthdays, or things like that. At work we also have a WhatsApp group of everything that is going on, if we have to give a message, it is given through there; if there is any information we have to give to clients, it is also sent through there. It's easier because it's just sending a message and that's it.” (FG^a^ 2)
	Facebook		“I agree with the others. I would prefer to use Facebook, which is how we can invite our family and friends. Remember that we have our families in our countries, and they are also on our Facebook. Everything that we give them to share, they can also see it. So I think that one of the platforms for us would be Facebook and I know that for the young people, it would be another and a very good one because some of the young people are not on Facebook anymore but on something else.” (FG 4)
**Delivery frequency and format**			
	Frequency		“As a journalist, I sell commercials. People get annoyed to hear your daily message, especially if you’re running the same thing every day. So, I think maybe every third day or two three times in a seven-day week period, to try not to annoy people with the same daily thing; in terms of the message if we’re trying to inform them of something, it's something that we want to let them know exists, but also not annoy them to a point where they don’t want to continue watching because it’s almost like a YouTube commercial, you don’t want to be pressing next every time. We want it to be there, but we don’t want it to be making you angry, that every time it’s the same message. We want to give you the information to remind you every once in a while about what's going on.” (FG 4) “As long as you can make the information just easy to understand. You know shorts and something that they could do every day. because if you're telling me that all they have, all they would have to do is put on a hat when they're going outside. I think people would be willing, as well as it doesn't interfere with your everyday activity or you're not adding no extra to their already schedules. And I think they would be willing to adhere to it.” (Interview 2)
	Format		“Short videos “I think what would also help if this program releases like simple everyday short videos, because we always see a lot of ads. So if you know, if we're going to be stuck. Haven’t seen an ad, if we see an ad that promotes, that’s telling me about health issues, but like very simple and direct, because I think a lot of people on their phone. They didn’t see these videos once in a while. So at least definitely videos that’s talked about the facts, or one about consequences, one about tips.” (FG 3) Visuals: “I think people are very responsive to like visual presentation. So maybe a drawing, and like some cues. Because then, that’s something easy to you know, with WhatsApp. When you send an image that almost automatically saves to your camera roll. And so, it’s something easy to forward too.” (Interview 2) “A lot of them don’t know how to read and write. I gather from my clients here, who I serve. So they’re more of a visual. They like to see videos if it was in that case. But they’re I don’t want to say lazy. They’re just, you know. They just don’t want to read.” (Interview 8)
**Message content and source**			
	Personalized messages		“The way of living in Puerto Rico is very different from the United States; Guatemala is another world; Mexico is another world. So, if I go to Puerto Rico, the sun is very strong, so I should look for more information on how to take care of myself in this type of environment. Here in the United States, things change a lot, and I think that maybe doing things depending on where the community is, maybe bringing things from each country, maybe teaching backgrounds of different people, or if the person is from Puerto Rico like me, what happened? Why did this happen to me when I lived there? I don’t know, things like that, if I’m understanding the question.” (FG 2)
	Personal stories and narratives		“Another thing would be the podcast, telling stories; people love stories, and they like that one because Latinos like drama and soap operas and that kind of thing, if it is a story of a case and the person is there telling what happened to him, people are interested, but also, if it is a long time, you lose people.” (FG 2) “I feel like some kind of personal testimonials.” (Interview 7)
	Cultural perspectives		“We are so very, very social culture. In the sense of, we’d like to be out and about in the summer, sun exposure, wherever we get it, you know, whether it be beach and, and going on vacation… maybe we talk to their neighbors and their neighbor, kids, and they all go socialize. So the socialization piece is important. The sense of community is important. And belonging is important.‬‬” (Interview 9)‬‬‬‬‬‬‬‬‬‬‬‬‬‬‬‬‬‬‬‬‬‬‬‬‬‬‬‬‬
	Message source		“So if you can get a Spanish speaking doctor. To talk to like about this is why we want you to do this. That might help, too, like somebody in a white coat. Okay this will impact the authority with knowledge. And they, you know, they have. They’ll have. They might have more respect. Listen more when the message is coming from someone in a white coat.‬” (Interview 3)‬‬‬‬‬‬‬‬‬‬‬‬‬‬‬‬‬‬‬‬‬‬‬‬‬‬‬‬‬‬‬‬‬‬‬‬‬‬‬‬‬‬‬‬‬‬‬‬‬‬‬‬‬‬‬‬‬‬‬‬‬‬‬‬‬‬‬‬‬‬ “I will say, a community leader, they probably would trust more, you know, in the work that we do is, but we do a lot of work in the African American community. And I know there’s a lot of a mistrust in the medical system. And for us, you know, who do we reach out to is to pastors, right, that that community leader, who has gained the trust of that community, to be able to speak on behalf of whatever it is that is helpful to you that we’re trying to promote‬” (Interview 9)‬‬‬‬‬‬‬‬‬‬‬‬‬‬‬‬‬‬‬‬‬‬‬‬‬‬‬‬‬‬‬‬‬‬‬‬‬‬‬‬‬‬‬‬‬‬‬‬‬‬‬‬‬‬‬‬‬‬‬‬‬‬‬‬‬‬‬‬‬ “They do want to be part of the decision-making process. They do also trust the medical providers.‬” (Interview 9)‬‬‬‬‬‬‬‬‬‬‬‬‬‬‬‬‬‬‬‬‬‬‬‬‬‬‬‬‬‬‬‬‬‬‬‬‬‬‬‬‬‬‬‬‬‬‬‬‬‬‬‬‬‬‬‬‬‬‬‬‬‬‬‬‬‬‬‬‬
**Engagement activities**			
		—^b^	“I like the idea, it will be like a buddy so they can help each other and kind of write each other out, and kind of have that that camaraderie like they’re going through with this together. And they have each other’s support. So I really like the idea of it as a group.” (Interview 3) “I think and focus on women. I mean that, and that’s true. Again, in any culture you get the women involved, and they’ll give the pressure to the to the man, and they’ll be the ones to take care of the children. And Latinos, I feel like, are much more open to these kinds of educational campaigns. They’re really the drivers. More so even than in other communities that I’ve dealt with. So yeah. I think I would focus on Latinos particularly.” (Interview 7)

^a^FG: focus group.

^b^Not applicable.

### Evaluation Outcomes

#### Intervention Platform

There was consensus that using social media was ubiquitous among the Hispanic community. They identified popular social media sites used by Hispanic individuals, with WhatsApp and Facebook platforms mentioned most frequently. When asked which social media site would be the most appropriate digital media platform to deliver a skin cancer intervention, ‬‬the ‬‬participants suggested that this would depend on the generation. As 1 participant from focus group 5 stated, “I would say that boomers and Gen X are very Facebook heavy, like it’s insane I would say that my generation does rely a little bit more on Instagram.”‬‬‬‬‬‬‬‬‬ However, these discussions mainly highlighted both Facebook and WhatsApp, with the latter being popular for some due to its “international reach, [which] is very popular” (focus group 5).‬‬‬‬‬‬‬‬‬

#### Delivery Frequency and Format

Participants were asked to identify the frequency of message delivery and format of information and messages for the intervention. The discussions regarding message delivery frequency ranged from every day to once or twice per week. Both community leader or health care provider participants and community members mentioned that short videos, visuals (eg, images and photographs), and simple texts messages would be effective and useful. A community leader suggested that a 3-month duration would be suitable for the intervention: “But the three months come and go more easily, which many people know how to do. Sending messages to each other is easy” (interview 11).

#### Message Content and Source

Recommendations for the content of health messages included having personalized messages, personal stories or narratives, design from Hispanic cultural perspectives and values, and potential trusted sources from the community. Some participants suggested that the use of visuals (eg, images and photographs) with primarily Hispanic individuals and people of color to help deliver the messages would be more appropriate to the community. One participant stated that “I guess when they do sunscreen, maybe they should have more people of color on there when they show skin cancer, maybe it would apply to us. Maybe we would feel like, okay, this happened to this Spanish person or this brown person, and then maybe we would be more likely to do it” (focus group 1). Other participants also mentioned that personal stories of skin cancer survivors and the use of narratives would be better to draw people’s attention and deliver the information. For example, 1 participant stated that “we do not all have the same skin, and we do not all have the same resistance to certain products, so we should listen to opinions and stories of other people who have gone through cancer or who have had this problem and let them explain what they would have done better, let them tell you what they did; examples of people who have had skin cancer and that they can discuss it with all the people” (focus group 2).

In addition, many participants mentioned that they came from different countries in Latin America, some with distinct norms surrounding skin tone and health behaviors. As such, showcasing a diverse group of Hispanic individuals to highlight the heterogeneity within the Hispanic community would be inclusive and a good strategy for diverse message design. One gave a detailed example: “We talked earlier about landscaping; we need people from the community. There are Guatemalans, Puerto Ricans, Mexicans, etc., who are working in that sector, to train them, explain the situation, the subject, and look for ways in which they could cooperate to create these informative materials because people have to see the importance of this in that labor sector. It could be a person from Colombia with a Colombian costume, a Mexican with a Mexican costume” (focus group 2).

Furthermore, some participants highlighted that community leaders would be a good source for health information in messages, as they have a trusted role in the community. Meanwhile, other participants mentioned that Hispanic individuals oftentimes comply with recommendations from health care providers and suggested that they would be appropriate message sources. For example, participants from our focus groups mentioned these points: “It will help more to have trusted people or trusted organizations to vouch for that” (interview 5) and “So, speaking of trust, it’s easier for someone I know or who looks like me to tell me something, and I pay attention to it, versus another type of person, physically speaking, even the language. I think culturally speaking, the cultural expression at this time, the information about symptoms, about...the process for self-examination, preventive measures, places to go, is already simplified, you just have to work with the affected communities of different ethnicities to record the message and make the educational materials” (focus group 2). Finally, many participants mentioned that they learned about sun protection behaviors from their mothers and suggested that we could target specific roles within the family or community, and that social interaction may play a significant role in influencing Hispanic individuals’ behaviors: “Moms are focused on getting home and preparing like normally it’s for their own good. It’s for the own good of their family, for their own kids. And showing them that [sun protection] is something they can easily do at home or at work or at school” (interview 10).

#### Fostering Engagement and Retention

Participants discussed what types of strategies and activities could increase engagement with the intervention and aid in retention: for example, having small incentives at different stages could increase engagement in the intervention; having testimonials from cancer survivors from the community might be motivational; keeping things short and engaging people on a regular basis; and trying not to “annoy people with the same daily thing.”

## Discussion

### Principal Findings

This study provides insights regarding whether Hispanic individuals at risk for skin cancer would be interested in a mobile-based intervention and what their suggestions are for intervention design. They showed interest in participating in a mobile-based skin cancer prevention intervention for the Hispanic community.

It is notable that participants ranged from young adults to older adults and both sexes. The intervention will target adult Hispanic individuals of both sexes to help broadly disseminate useful and relevant health messages to expand intervention effects on skin cancer prevention behaviors among Hispanic individuals.

Hispanic participants in our study had consensus that using social media would be promising due to a large number of Hispanic individuals being social media users. Among the social media platform themes emerging from our focus group interviews and community leader interviews, WhatsApp was a widely used platform across different age groups. Participants mentioned using WhatsApp to communicate among family and friends using both groups and private messages frequently, which provide insights on delivering customized skin cancer prevention messages through different message formats and audiences. These findings are consistent with literature regarding social media among US Hispanic individuals, which report Hispanic individuals using WhatsApp at the highest rates and as a way to communicate with friends and family internationally [28,29].

It was determined that the use of succinct video clips and visual illustrations, where Hispanic presenters play a pivotal role, in conjunction with the presentation of statistics concerning skin cancer prevalence among Hispanic individuals, holds significant potential in affecting behavioral change and disseminating knowledge about skin cancer within this demographic. Participants expressed diverse preferences regarding the frequency of intervention delivery; nevertheless, there was a consensus that it should not be overly frequent.

Customized messages, personal stories and narratives, and messages designed based on Hispanic cultures or values were repeatedly mentioned. These findings reinforced the importance of a culturally relevant and community-engaged approach in designing health interventions for Hispanic individuals [21,22]. Hispanic individuals are known to place a substantial emphasis on the role of family [30] and tend to trust community leaders or organizations they have connections with. Hence, it was proposed that focusing on educating women within Hispanic families on sun safety practices could yield cascading benefits for the entire household. Furthermore, given the robust ties that Hispanic individuals maintain with community leaders and the presence of Hispanic-specific organizations, it was collectively agreed that the involvement of these local Hispanic entities in the intervention would serve to bolster its credibility and reach [31].

### Limitations

Our study has a few limitations. First, our study used a mix of individual interviews of community leaders and health care providers, as well as group interviews among Hispanic community members; however, the focus group format may have generated a bias due to the group influence of the discussions versus individual interviews, where an individual’s perspective may have skewed the opinions of others participating in the focus group interview. Second, the Zoom video camera feature was highly encouraged but not required for participation; therefore, some focus group participants chose not to have their videos on. This may have potentially limited opportunities for rapport building and observing nonverbal cues. Third, interviews were conducted via Zoom videoconferencing to facilitate participation, thereby mitigating constraints related to time and geographical distance. However, this may limit participation of those who do not have access to the internet or more sophisticated technical skills. Our sample also comprised a significant proportion of Hispanic individuals with college degrees. In addition, the eligibility criteria (wearing long-sleeved shirts and long pants in the last 3 months) may be impacted by our data collection time (starting from March to July), which could potentially exclude participants who do not engage in sufficient sun protection behaviors in the summertime. Since diffuse UV can be high in winter [32,33] and some Jersey shore areas had UV index above 3 starting from February 2023 [34], it would be important to weigh these factors (eg, seasons and latitudes) for eligibility and recruitment in skin cancer prevention. Future studies should take these factors into consideration to build a more robust recruitment plan and interview process. Furthermore, the next step of our intervention design will include more comprehensive information about UV index and sun protection in all seasons.

### Conclusions

Our study engaged both community members and key stakeholders to offer their perspectives in designing a digital skin cancer prevention intervention targeting the Hispanic community for skin cancer prevention behavior change. The findings from community leaders and health care providers offered meaningful insights that complemented the findings from the community. Using WhatsApp or Facebook, including short videos, visuals, simple text messages, personalized messages, personal stories and narratives, culturally relevant design, and community-trusted sources, would be potentially effective for the digital skin cancer prevention.

## Abbreviations

COE: Community Outreach and Engagement

## Data Availability

The qualitative data sets from this study are not publicly available due to privacy issues but are available from the corresponding author on reasonable request.
